# Effect of Prophylactic Phenylephrine Infusion Versus Interventional Ephedrine Boluses on Umbilical Blood pH in Cesarean Deliveries Under Spinal Anesthesia: A Retrospective Case-Control Study

**DOI:** 10.3390/jcm14176016

**Published:** 2025-08-26

**Authors:** Bartosz Horosz, Katarzyna Białowolska-Horosz, Małgorzata Malec-Milewska

**Affiliations:** Department of Anesthesiology and Intensive Care, Centre of Postgraduate Medical Education, Orlowski Hospital, 00-416 Warsaw, Poland

**Keywords:** ephedrine, phenylephrine, spinal, hypotension, acidosis

## Abstract

**Background/Objectives:** Hypotension is a common complication of spinal anesthesia for cesarean section. Although phenylephrine has replaced ephedrine as the first-line vasopressor, comparative data on neonatal outcomes remain important in clinical decision-making. The objective of this study was to compare the effects of prophylactic phenylephrine infusion versus interventional ephedrine boluses on umbilical artery pH and maternal hemodynamic stability in women undergoing cesarean section under spinal anesthesia. **Methods:** In this retrospective case-control study we analyzed perioperative and neonatal data of elective cesarian section cases where either ephedrine boluses (total dose of more than 15 mg) or prophylactic phenylephrine infusion were employed for blood pressure control following spinal anesthesia. Demographic, hemodynamic, obstetric and neonatal data were extracted from medical records. Ninety-four elective cesarean section cases were included. Umbilical artery pH, base excess, and Apgar scores were assessed as primary and secondary neonatal outcomes. The lowest recorded systolic blood pressure (SBP), mean arterial pressure (MAP), and incidence of nausea and vomiting were evaluated as maternal outcomes. **Results:** Umbilical artery pH and other blood gas parameters did not differ significantly between groups. Neonatal acidosis (pH < 7.2) occurred in two cases in the ephedrine group while none were noted in the phenylephrine group. Maternal hemodynamic stability was significantly better in the phenylephrine group, with higher nadir SBP and MAP (*p* < 0.001). Nausea was more common with ephedrine (42.5% vs. 10.6%, *p* < 0.001), and vomiting occurred only in this group. **Conclusions:** Prophylactic phenylephrine infusion provides superior maternal hemodynamic stability and better tolerance during cesarean delivery compared to interventional ephedrine boluses, without change in neonatal acid–base status.

## 1. Introduction

The prevention and management of hypotension resulting from sympathetic blockade due to spinal anesthesia have been the subject of ongoing investigation for many years, particularly in the context of regional anesthesia for cesarean delivery [1].

Ephedrine, an indirectly acting sympathomimetic with some direct effects, and phenylephrine, a selective α1-adrenergic receptor agonist, are currently the two most frequently used pharmacologic agents for this purpose. Traditionally, ephedrine was used to prevent and treat hypotension associated with neuraxial anesthesia, as there was concern that pure alpha-adrenergic agonists (e.g., phenylephrine) could reduce uterine blood flow [2]. However, ephedrine use has been associated with an increased risk of maternal tachycardia and adverse effects on fetal acid–base balance [3]. More recent clinical data have not confirmed a detrimental impact of direct-acting vasopressors in this setting, and phenylephrine has been shown to offer more favorable maternal hemodynamic stability than ephedrine [3]. Moreover, emerging evidence has suggested a potentially more favorable fetal profile with phenylephrine use [4,5]. As a result, phenylephrine is now recommended as the first-line agent for spinal anesthesia-induced hypotension during cesarean section, despite its known association with an increased risk of maternal bradycardia and decreased cardiac output [6,7].

Numerous studies have compared the use of ephedrine and phenylephrine for both the prevention and treatment of hypotension during cesarean delivery [6,8]. Following the implementation of phenylephrine as the vasopressor of choice in our center, we conducted a retrospective study to compare maternal and fetal outcomes between the use of prophylactic phenylephrine infusion and interventional boluses of ephedrine for the management of spinal anesthesia-induced hypotension during cesarean delivery.

## 2. Materials and Methods

This retrospective analysis was approved by the Ethics Committee of the Centre of Postgraduate Medical Education (CMKP) on 13 November 2024 (96/2024), and registered at ClinicalTrials.gov (NCT06741410).

### 2.1. Data Collection

We reviewed the medical records of all patients who underwent elective cesarean delivery between 2020 and 2022 at Orlowski Hospital, CMKP, Warsaw, Poland. During the initial screening process we identified obstetric cases meeting the following inclusion criteria: maternal age > 18 years, BMI < 40, ASA status < 3, and elective cesarean section under spinal anesthesia at term (>37 weeks gestation). Further analysis of medical notes aimed to identify 50 cases where more than 15 mg of ephedrine was used to treat maternal hypotension (ephedrine group) and 50 cases where continuous prophylactic infusion of phenylephrine was used for control of blood pressure (phenylephrine group). Cases with diagnosed pregnancy-induced hypertension (PIH) and known fetal abnormalities were excluded. We included cases in a consecutive manner until the required number in each group was reached.

Maternal demographic and pregnancy-related data were acquired from medical records. Anesthetic records were reviewed for hemodynamic and anesthesia-related variables. Umbilical artery blood gas values were obtained from the hospital’s laboratory electronic database and 5 min Apgar scores from neonatal medical records.

All patient information was anonymized, and the requirement for informed consent was waived by the Ethics Committee due to the retrospective nature of the study. Measures were taken to ensure that no patient could be identified from the data used in the research. Additionally, all identifiable data utilized during retrieval and analysis were strictly accessible only to the investigators.

### 2.2. Spinal Anesthesia Technique and Management

Spinal anesthesia for elective cesarean section is standardized in our center. Parturients are required to fast for 6 h before surgery. Crystalloid preloading is used (1000 mL within one hour prior to procedure), as well as ranitidine and metoclopramide for prevention of aspiration-related pneumonia.

After admission to the operating room, parturient routine monitoring is commenced, which involves the following measurements: 3-lead ECG, pulse-oximetry and automated noninvasive blood pressure (NIBP). Blood pressure is measured twice prior to spinal injection and the lowest of the two readings is considered baseline. The interval for NIBP measurement is set at 3 min. Co-loading with crystalloid fluid is commenced with the goal to infuse 250 mL before spinal anesthesia. Spinal injection is preferably done in sitting position, with 10 to 12.5 mg of hyperbaric bupivacaine injected into L3/4 or L4/5 interspace under aseptic conditions. Left lateral tilt is used for venous return optimization.

Assessment of the spinal block dynamic is done every 1 min following spinal injection for the first 10 min and then every 3–5 min. Sensory loss to cold is tested with a gauze soaked in alcohol-based aseptic solution or ethyl chloride spray in the mid-clavicular line. A sensory block of at least T6 is required for surgery to start.

The highest recorded level of sensory blockade and the intrathecal dose of hyperbaric bupivacaine were obtained from the records and used for analysis.

### 2.3. Hypotension Management

Ephedrine was routinely administered in intravenous boluses of 5 mg. The decision regarding the use of ephedrine and the total number of doses administered was determined by the anesthesiologist responsible for the conduction of the anesthesia.

Following the introduction of phenylephrine into our clinical practice, and during the course of this study, it was routinely administered in our center according to a standardized protocol. In cases that were planned to be managed with the use of phenylephrine it was prepared at a concentration of 100 mcg/mL and delivered via continuous intravenous infusion initiated immediately after the spinal injection. Initial infusion rate was set at 33 mcg/min (20 mL/h), and adjustments were made by either doubling or halving the rate as needed, based on the discretion of the anesthesiologist. The target was to maintain systolic blood pressure (SBP) above 100 mmHg. In cases where the hemodynamic effect was insufficient despite an increased infusion rate, boluses of 50 mcg of phenylephrine (0.5 mL) were administered. If SBP rose above 20% of baseline, infusion was to be withheld and restarted at 20 mL/h if it dropped below baseline again. Bradycardia (HR < 60/min) was managed with a bolus of 0.5 mg atropine, irrespective of SBP. Lowest values of SBP and mean arterial blood pressure (MAP) recorded up to 30 min following spinal incision were acquired for analysis, as well as episodes of bradycardia, nausea, and vomiting.

As a routine, a neonatal blood gas sample was acquired from the umbilical artery of the double-clamped segment of the umbilical cord by the obstetrician performing the surgery. Neonatal assessment after birth was done and recorded in medical records by the neonatologist that was always present in cases of operative delivery.

### 2.4. Statistical Analysis

The study was designed to detect a difference in umbilical blood pH between the ephedrine and phenylephrine groups as a primary outcome measure. The sample size calculation for a test power of 80% and α = 0.05 (two-sided) was performed using literature data on the average pH and the expected difference between groups. Forty-four patients in each group were required to detect the difference of 0.03 with the assumption of an average pH of 7.28 ± 0.05 [9,10]. To allow for potential drop-outs, a total of 50 cases was to be included in both groups. Secondary outcomes were base excess and hemodynamic data.

Student’s unpaired t-test was used to compare normally distributed continuous variables and a Mann–Whitney (Wilcoxon) W-test was used for for continuous variables with non-normal distribution. Normality was tested using standardized skewness and kurtosis of the data, which if outside the range of −2 to +2 indicated a significant departure from normality. Fisher’s exact test was employed to compare categorical data. For the purpose of data analysis, the level of maximal sensory blockade was converted into numbers (1–6 for levels T5–C8, respectively). A significance threshold of *p* < 0.05 was applied to all tests. All analyses were performed with the use of Statgraphics Centurion XVII software.

## 3. Results

A manual review of medical records was conducted to identify 50 consecutive cases in which more than 15 mg of ephedrine was used to treat hypotension, and 50 consecutive cases in which phenylephrine infusion was administered for the prevention and treatment of spinal block-related hypotension. Following a thorough analysis of these 100 cases, data from 94 parturients (47 in each group) and 97 neonates (48 in the ephedrine group and 49 in the phenylephrine group) were found to be complete and included in the final analysis. The demographic characteristics, obstetric data, and spinal anesthesia-related parameters are presented in Table 1.

The groups were comparable in terms of general demographics. However, gravidity and parity were significantly higher in the ephedrine group (*p* = 0.009 and *p* = 0.045, respectively). Neonatal birthweight was also significantly greater in the ephedrine group.

Preoperatively, mean systolic blood pressure was similar between groups. However, baseline systolic blood pressure was lower, and the average dose of bupivacaine was higher, in the ephedrine group. The median dose of ephedrine administered was 25 mg, while the median dose of phenylephrine infused was 720 mcg.

**Figure 1:**
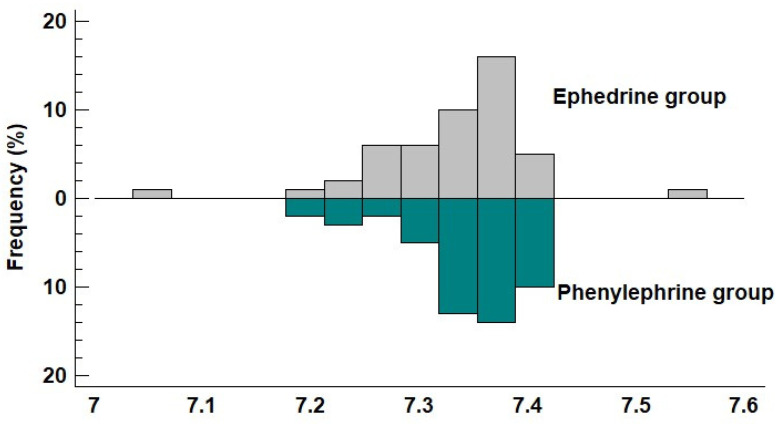
Frequency histogram of pH **values** in ephedrine and phenylephrine groups.

**Figure 2:**
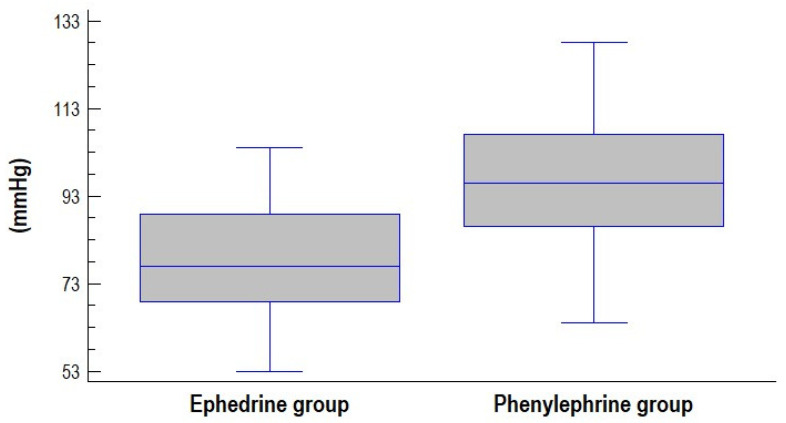
Box and whisker diagram of lowest recorded systolic blood pressure (SBP) in groups assessed.

**Figure 3:**
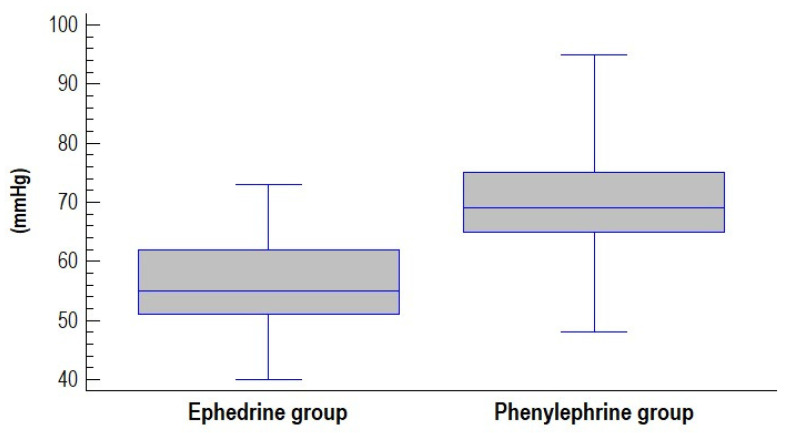
Box and Whisker diagram of lowest recorded mean arterial pressure (MAP) in groups assessed.

No significant differences were observed between groups in umbilical cord blood pH (Figure 1) or in other parameters of umbilical blood gas analysis (Table 2). A pH value < 7.2 was recorded in only two cases, both in the ephedrine group (5-min Apgar scores were 8 and 10). Mean values of the lowest recorded systolic blood pressure (SBP) and mean arterial pressure (MAP) were significantly lower in the ephedrine group compared to the phenylephrine group (Figure 2 and Figure 3).

Nausea occurred significantly more frequently in the ephedrine group, and vomiting was reported exclusively in this group. Bradycardia was rare, as it occurred in only 2 cases in the ephedrine group and in 5 cases in the phenylephrine group. The difference was not statistically significant.

## 4. Discussion

This retrospective case-control study aimed to compare the effect of prophylactic phenylephrine infusion versus interventional ephedrine boluses on neonatal umbilical artery pH and maternal hemodynamic stability in women undergoing cesarean delivery under spinal anesthesia. The findings suggest that while both vasopressors resulted in comparable neonatal acid–base status, phenylephrine was associated with improved maternal hemodynamic control and a lower incidence of intraoperative nausea.

Previous studies have established that hypotension following spinal anesthesia is nearly universal without vasopressor support, and that it may compromise uteroplacental perfusion, leading to neonatal acidosis [11,12,13]. Prophylactic administration of phenylephrine has emerged as a preferred strategy for maintaining maternal blood pressure and optimizing neonatal outcomes [6,12].

Umbilical artery pH and other blood gas values did not differ significantly between the two groups in our study, despite these hemodynamic differences. The results were similar for the Apgar scores. It appears that changing the choice of vasopressor did not result in a change of fetal acid-base status in our institution. Historically, higher values of umbilical pH in phenylephrine groups were reported by the prospective trials that compared ephedrine and phenylephrine use in elective cesarean sections, with a weighted mean difference of 0.03 to 0.06 [7,14]. Whether a difference of this magnitude is clinically significant remains a topic of debate. It was suggested in the past that the threshold value of pH for unfavorable neonatal outcomes is 7.2. More recent data indicate that the lower limit of a normal range of umbilical blood pH at birth could be as low as 7.0 [15]. It is argued that the difference between the use of ephedrine and phenylephrine is most likely attributed to greater transfer of ephedrine than phenylephrine through the placental barrier and its less rapid metabolism in the fetus. It allows for fetal β-receptor stimulation, an increase in fetal metabolic activity, and a consequent rise in the acidic components of the acid–base equilibrium [5]. The fact that in our cohort no difference in pH was detected between the groups may be contributed to many factors, among others the limited dose of ephedrine used and bolus mode of administration. Although the median dose of administered ephedrine was 25, most of the trials that reported significant pH differences employed vasopressor infusions with much higher total doses of ephedrine [5,16,17]. In fact, there are numerous reports pointing towards similar umbilical acid–base status, especially when boluses of ephedrine were used and compared to phenylephrine administration [18,19,20]. In a randomized study by Nazir et al., no differences in umbilical pH and Apgar scores were found between ephedrine and phenylephrine groups when both vasopressors were used as prophylactic and interventional iv boluses [19]. They were also similar in terms of maternal blood pressure control. Bhardwaj et al. compared the blood pressure values and neonatal outcomes in parturients receiving prophylactic infusions of ephedrine, phenylephrine, and metaraminol following spinal anesthesia for cesarean section. No significant differences were reported in umbilical acid–base status and Apgar scores, while the difference in mean pH did not exceed 0.02 between the three groups [20]. The mean dose of ephedrine until delivery was nearly 40 mg in this study. In the study by Soxhuku-Isufi et al., bolus doses of ephedrine and phenylephrine were used. Although the mean umbilical blood pH was statistically lower in the ephedrine group, the difference was only 0.02, and no other differences in neonatal or maternal outcomes were observed [18].

Similar findings have consistently been reported in studies involving non-elective cesarean section cohorts [10,21,22,23]. In cases of potential fetal compromise, the choice between ephedrine and phenylephrine does not appear to influence neonatal acid–base status or Apgar scores. In a retrospective analysis, Cooper et al. compared the acid–base status of neonates born before and after a change in the routinely used first-line vasopressor for urgent cesarean deliveries [8]. They found no significant differences in pH values or in the incidence of pH < 7.2. Similar results were observed in a subsequent analysis by the same research group, which also included urgent cesarean deliveries in which no vasopressor was administered. No significant differences in umbilical pH or the incidence of acidosis were found among the ephedrine, phenylephrine, and no-vasopressor groups. Overall, no association was identified between the type of vasopressor used and neonatal acid–base status [21].

In our cohort, the phenylephrine group exhibited a significantly higher nadir SBP and MAP compared to the ephedrine group, which aligns with some prior evidence that phenylephrine offers superior control of maternal blood pressure [9,24]. Less strict control of blood pressure in the ephedrine group was not accompanied by derangement in umbilical acid–base outcomes. This finding is in line with other studies where it has been shown that the rate of neonatal acidosis was more likely related to ephedrine dose rather than the quality of hemodynamic control. In the randomized study involving elective cases of cesarean section, prophylactic ephedrine infusion offered better hemodynamic stability than phenylephrine infusion (average dose 34 mg in the first 15 min) and yet resulted in a much higher rate of acidosis (50% vs. 9%, respectively) [25]. In our study only two cases of pH < 7.2 were recorded, both in the ephedrine group (4.2%), and the difference between the groups was not statistically significant.

One of the most notable differences between groups in our study was the incidence of intraoperative nausea. Women who received ephedrine experienced significantly more nausea (42.5% vs. 10.6%, *p* < 0.001), a finding that has been reported in the literature [5]. This effect may be attributed to the more pronounced and rapid blood pressure fluctuations observed in the ephedrine group in our study, although similar findings have also been reported in studies where blood pressure control was equally effective in both the ephedrine and phenylephrine groups [5,26]. Vomiting was also noted exclusively in the ephedrine group, though overall frequency was low.

This study has several limitations. First, its retrospective design precludes causal inference and may introduce an obvious selection or documentation bias. Although consecutive cases that fulfilled the inclusion criteria were enrolled in both groups, they were not randomly assigned. Second, the sample size, while adequate for detecting moderate group differences and calculated to detect predefined differences in umbilical pH, may have limited power for rare neonatal outcomes such as pH < 7.2. Third, the compared hypotension management strategies differed in their modes of administration: a prophylactic infusion in the phenylephrine group versus interventional boluses in the ephedrine group. While this reflects the study design, which was aimed at comparing these two strategies, it is possible that the results might have differed if both vasopressors had been administered using the same mode (either as bolus or infusion). Finally, individual anesthetic management (e.g., dosing decisions, fluid co-loading strategies) was not protocolized and may have varied between providers.

## 5. Conclusions

In conclusion, prophylactic phenylephrine infusion during cesarean delivery under spinal anesthesia was associated with better maternal hemodynamic stability and a significantly lower incidence of intraoperative nausea compared to interventional ephedrine boluses. Both vasopressors provided comparable neonatal outcomes in terms of umbilical artery pH and Apgar scores. These findings support the routine use of phenylephrine as the first-line vasopressor in obstetric anesthesia to optimize both maternal comfort and neonatal safety.

## Figures and Tables

**Table 1:** Maternal demographics and procedure-related data. Data presented as geometric mean (standard deviation), median (first, third quartiles), or *n* (%). *—average ranks. Abbreviations: SBP—systolic blood pressure; MAP—mean arterial pressure; CI—confidence interval.

	Ephedrine	Phenylephrine	Standard Difference (95%CI)	*p*
N	47	47		
Age, years	33.80 ± 4.72	33.06 ± 4.74	0.74 (−1.19; 2.68)	0.447
Weight, kg	81.04 ± 11.75	78.74 ± 11.39	2.3 (−2.44; 7.03)	0.338
Height, cm	167.38 ± 6.32	165.80 ± 6.29	1.58 (−1.01; 4.16)	0.229
Weight gain, kg	13.92 ± 4.65	12.98 ± 4.69	0.93 (−0.97; 2.85)	0.334
Hemoglobin, g/dL	12.31 ± 1.10	12.27 ± 1.01	0.04 (−0.39; 0.47)	0.846
Gestational age (wk)	38.57 ± 0.82	38.42 ± 1.01	0.15 (−0.23; 0.52)	0.438
Twin pregnancies	1	2		
Gravidity	3.00 (4.00; 4.00)	2.00 (1.00; 3.00)	(54.61; 40.38) *	0.009
Parity	2.00 (1.00; 3.00)	2.00 (1.00; 2.00)	(52.81; 42.18) *	0.045
Baseline SBP (mmHg)	130.02 ± 13.83	136.76 ± 12.85	−6.74 (−12.21; −1.27)	0.016
Baseline MAP (mmHg)	99.91 ± 12.10	101.12 ± 11.39	−1.21 (−6.05; 3.62)	0.619
Bupivacaine dose (mg)	11.97 ± 0.91	11.14 ± 1.09	0.83 (0.42; 1.24)	<0.001
Lowest SBP	77.87 ± 12.36	96.76 ± 15.16	−18.89 (−24.56; −13.22)	<0.001
Lowest MAP	56.47 ± 8.25	69.97 ± 10.77	−13.50 (−17.46; −9.53)	<0.001
Total vasopressor dose (mg)	25 (20; 35)	0.72 (0.428; 1)		
Maximal upper sensory block level	T3 (T4; T2)	T2 (T1; T3)	−1 (−1; 1)	0.06
Crystalloids volume (mL)	1397.87 ± 311.02	1417.02 ± 312.3	−19.15 (−146.84; 108.54)	0.77
Newborn weight (g)	3540.52 ± 449.95	3331.53 ± 484.34	208.99 (20.45; 397.52)	0.03
Bradycardia	2 (4.2)	5 (10.6)	−6.4 (−16.9; 4.17)	0.435
Nausea	20 (42.5)	5 (10.6)	31.9 (15.3; 48.6)	<0.001
Vomiting	1 (2.1)	0 (0)	2.1 (−2.0; 6.3)	1.0

**Table 2:** Umbilical blood gases and neonatal outcomes. Data presented as median (first, third quartiles), or *n* (%). Abbreviation: CI—confidence interval.

	Ephedrine	Phenylephrine	Standard Difference (95%CI)	*p*
N	48	49		
pH	7.34 (7.29; 7.37)	7.35 (7.31; 7.38)	−0.008 (−0.03; 0.01)	0.396
PaCO_2_ (mmHg)	45.3 (41.75; 52.17)	46.5 (42.05; 52)	−0.54 (−4.12; 3.02)	0.899
PaO_2_ (mmHg)	24.75 (18.22; 30.3)	21.7 (17.8; 27.75)	1.90 (−2.35; 6.16)	0.310
Base excess (mEq/L)	−1.8 (−3.32; −0.6)	−1.4 (−2.5; 0.1)	−0.85 (−1.66; −0.05)	0.062
Umbilical artery pH < 7.2	2 (4.2)	0 (0)	4.2 (−1.47; 9.81)	0.24
Apgar score at 5 min	10 (10; 10)	10 (10; 10)	0 (0; 0)	0.94

## Data Availability

Source data related to this paper are available from the corresponding author on reasonable request.

## Abbreviations

The following abbreviations are used in this manuscript:

SBP	Systolic Blood Pressure
MAP	Mean Arterial Pressure
CI	Confidence Interval
HR	Heart Rate
PIH	Pregnancy-Induced Hypertension
